# Clinical value of endoluminal ultrasonography in the diagnosis of rectovaginal fistula

**DOI:** 10.1186/s12880-016-0131-2

**Published:** 2016-04-06

**Authors:** Hao-Qiang Yin, Chen Wang, Xin Peng, Fang Xu, Ya-Juan Ren, Yong-Qing Chao, Jin-Gen Lu, Song Wang, Hu-Sheng Xiao

**Affiliations:** Department of Ultrasonic Diagnosis, Longhua Hospital Affiliated to Shanghai University of Traditional Chinese Medicine, Shanghai, 200032 China; Department of Anorectal Surgery, Longhua Hospital Affiliated to Shanghai University of Traditional Chinese Medicine, Shanghai, China; Department of Radiology, Longhua Hospital Affiliated to Shanghai University of Traditional Chinese Medicine, Shanghai, China

**Keywords:** Endoluminal ultrasonography, Diagnosis, Rectovaginal fistula (RVF)

## Abstract

**Background:**

Rectovaginal fistula (RVF) refers to a pathological passage between the rectum and vagina, which is a public health challenge. This study was aimed to explore the clinical value of endoluminal biplane ultrasonography in the diagnosis of rectovaginal fistula (RVF).

**Methods:**

Thirty inpatients and outpatients with suspected RVF from January 2006 to June 2013 were included in the study, among whom 28 underwent surgical repair. All 28 patients underwent preoperative endoluminal ultrasonography, and the obtained diagnostic results were compared with the corresponding surgical results.

**Results:**

All of the internal openings located at the anal canal and rectum of the 28 patients and confirmed during surgery were revealed by preoperative endosonography, which showed a positive predictive value of 100 %. Regarding the 30 internal openings located in the vagina during surgery, the positive predictive value of preoperative endosonography was 93 %. The six cases of simple fistulas confirmed during surgery were revealed by endosonography; for the 22 cases of complex fistula confirmed during surgery, the positive predictive value of endosonography was 90 %. Surgery confirmed 14 cases of anal fistula and 14 cases of RVF, whereas preoperative endoluminal ultrasonography suggested 16 cases of anal fistula and 12 cases of RVF, resulting in positive predictive values of 92.3 and 93 %, respectively.

**Conclusion:**

The use of endoluminal biplane ultrasonography in the diagnosis of RVF can accurately determine the internal openings in the rectum or vagina and can relatively accurately identify concomitant branches and abscesses located in the rectovaginal septum. Thus, it is a good imaging tool for examining internal and external anal sphincter injuries and provides useful information for preoperative preparation and postoperative evaluation.

## Background

Rectovaginal fistula (RVF) refers to a pathological passage between the rectum and vagina and is also known as fecal fistula. The incidence of rectovaginal fistula worldwide was estimated to be two million in 2006 [1]. It has been estimated that just over one million women are afflicted in sub-Saharan Africa and south Asia alone, with approximately 6000 new cases yearly in these two world regions and 50,000 to 100,000 new cases annually worldwide [2]. In the United States, most of these fistulas are secondary to obstetrical injury, although a large constituent also spontaneously arises in the setting of Crohn’s disease. Neoplasms, infectious diseases, and operative and non-operative trauma are also contributory causes [3].

Due to the particularity and complexity of the local anatomy of the lesion, RVF often reduces the quality of life of affected women and represents a challenge for surgeons [4]. The vaginal passage of gas and stool can cause physical symptoms due to inflammation and irritation. Patients may also suffer from significant psychosocial and sexual dysfunction [5]. RVFs are classified according to their distance from the site of the hymen to the distal margin of the fistula as low, high and intermediate [6]. In low fistulas, the opening is near the posterior vaginal fourchette. In high fistulas, the opening is behind or near the cervix, whereas midrectovaginal fistulas are between the locations for the low and high fistulas [7].

The bimanual examination can generally confirm the presence of dimples that indicate RVF. With the help of anoscopic or speculum examination, the size and location of the fistula can be detected [8]. If the diagnosis remains elusive, filling the vagina with water and looking for air bubbling during rigid sigmoidoscopy can be useful to determine the location of RVF. When thorough physical examination and examination under anesthesia fail to localize a fistula of high clinical suspicion, specialized diagnostic imaging is needed [9]. Standard endoanal ultrasonography can be used to locate internal openings and define the sphincter anatomy of anorectal fistula and RVF with a 7 to 73 % accuracy [10–12]. The addition of hydrogen peroxide contrast injected into the tract increases the diagnostic accuracy to approximately 48 to 73 % [10–12]. However, few studies have been reported using bi-probe diagnosis combined with transrectal and transvaginal ultrasonography. Here, the preoperative endoluminal ultrasonography results of 28 RVF patients were compared with the corresponding surgical findings to explore the diagnostic value of RVF by endosonography and help facilitate the clinical diagnosis of RVF.

## Methods

### Ethics statement

Written informed consent was obtained from all of the patients for publication of this article and any accompanying images. All patient gave written informed consent to participate in the study. This study was approved by the Ethics Committee of Longhua Hospital Affiliated with the Shanghai University of Traditional Chinese Medicine.

### General information

From January 2006 to June 2013, 34 inpatients and outpatients at Longhua Hospital, Shanghai, China, with an average age of 44.5 years (range: 29–68 years) had suspected RVF. During the same period of time, 5086 cases of anal fistula were treated at Longhua Hospital. Thus, the proportion of the patients with suspected RVF was approximately 6/1000. Of these 34 patients, 28 underwent surgical repair. For the six patients who did not undergo surgery, three were transferred to the medical department for the treatment of Crohn’s disease, one was a cancer patient, and two missed the opportunity for surgical treatment due to birth trauma. In all 28 patients, RVF was mediated through acquired factors, including perianal abscesses in ten cases, anal cryptitis and secondary abscesses in seven cases, Bartholin’s gland abscesses in five cases, traumas in four cases, and birth traumas in two cases.

### Equipment

HDI 5000-X RES color Doppler ultrasonography (Phillips, The Netherlands) and the biplane transrectal probe BP10-5ec were used. The diameter of the probe was 19 mm; the frequency of the convex array probe was 5–10 MHz; the imaging angle was 150°, and the frequency of the linear array probe was 5–12 MHz. During the examination, a condom was placed over the surface of the probe.

### Ultrasonography examination

Prior to the examination, no special patient preparation was needed. The patients were instructed to assume a left-side supine knee-chest position, and the probe was gently inserted into the rectum or vagina and then slowly retracted. The pelvic floor muscles at the lower rectum, and the internal and external anal sphincters surrounding the anal canal were identified. According to splinters identifiable by endosonography signals, the anorectum was divided into the following three sections: the upper section was the part of the rectum surrounded by the puborectal muscle; the middle section was the part of rectum surrounded by the deep and superficial layers of the external anal sphincter and anal canal; the lower section was the anal canal surrounded by the subcutaneous part of the external anal sphincter. The lower rectum and anal canal were carefully examined. The direction of the fistula and its relation to the sphincter and levator ani muscle, as well as the location of the internal positions, were recorded in detail. The length and diameter of the main branch of the fistula were measured, and the type of fistula was determined based on the position of its internal opening in the anal canal or rectum. If the internal opening of the fistula was located at or below the dentate line, it was identified an anal-vaginal fistula. If the opening was located above the dentate line, it was identified as RVF. In addition, the fistulas were divided into the following two categories based on their morphology: simple and complex fistulas. For a simple fistula, the opening to the vagina directly connects to the opening of the anorectum, and the shape of the fistula is typically a straight line. For complex fistula, the opening to the vagina not only connects to the opening of the anorectum but also connects to the perianal fistula, perineal subcutaneous fistula or fistula deep in the rectovaginal septum, typically creating a “Y” or “T” shape. Complex fistulas also include combined branches or abscesses extending to the rectovaginal septum or branches or abscesses extending to perianal or perineal regions. If the imaging was not satisfactorily clear, 5–10 ml of hydrogen peroxide or saline was injected from the subcutaneous external opening of the fistula, and new probes were used. Transrectal or transvaginal cross-sectional scanning using convex array probes and vertical section scanning using linear array probes were performed. All of the images and videos obtained were stored in the ultrasonography workstation for subsequent off-line retrospective study. Two experienced sonographers independently read and evaluated the images. All of the ultrasonography examination results were compared with the surgical findings. Correct descriptions of the internal openings must include the correct quadrant (within 60°) and correct positional level (anal or rectal). Correct descriptions of the main branch must include the correct number, location and direction, and correct descriptions of secondary branches or abscesses must include the correct number, location and direction toward the rectovaginal septum or perianal or perineal regions, and the size, and general shape must be described as a straight line or “Y” shaped.

### Statistical analysis

All of the sonograms were systematically and independently reviewed by two sonographers. The kappa consistency test was performed to evaluate interobserver variability. After calculating the κ value, if a disagreement still existed, the same two observers reevaluated the images together until consensus was reached. For comparison with the surgical findings, the positive predictive value and sensitivity of endoluminal ultrasonography were calculated in diagnosing the fistula morphology, internal openings and abscesses.

## Results

There was complete agreement between the observers for all items on the evaluated sonograms, except for the presence of secondary perianal fistulas or other extensions (agreement, 91 %; k, 0.75). The fistula opening on the vaginal side had a diameter of 2.2–7.7 mm, with a mean value of 3.3 mm. The opening on the rectal or anal side had a diameter of 1.5–5.2 mm, with a mean value of 2.8 mm. The main branch of the fistula had a length of 11–32 mm, with a mean value of 23.5 mm. All of the internal openings were located at the anal canal, and the rectums of the 28 patients were revealed through preoperative endosonography, showing a positive predictive value of 100 %. Surgery confirmed 30 internal openings located in the vagina, and preoperative endosonography also revealed 30 openings, including 28 true-positive cases, two false-positive cases, and two false-negative cases, resulting in a positive predictive value of 93 %. All six cases of simple fistulas confirmed during surgery were revealed by endosonography. For the 22 cases of complex fistulas confirmed during surgery, preoperative endosonography readings indicated 18 true-positive cases, two false-positive cases, and two false-negative cases, resulting in a positive predictive value of 90 %. Endoluminal ultrasonography also revealed all five cases of concomitant rectovaginal septum abscesses. The surgeries confirmed 14 cases of anal fistulas and 14 cases of RVF, whereas preoperative endosonography suggested 16 cases of anal fistulas and 12 cases of RVF, resulting in positive predictive values of 92.3 and 93 %, respectively (Table 1).

**Table 1:** Diagnostic accuracy of 28 cases of anal fistulas using endoanal ultrasound

Confirmed morphology	Endosonography finding	Surgical finding	Positive predicative value	Sensitivity
Simple fistula	6	6	100 %	100 %
Complex fistula	20	22	90 %	90 %
Opening to vaginal wall	30	30	93 %	93 %
Opening to anal canal or rectum	28	28	100 %	100 %
Abscess	5	5	100 %	100 %
Anal fistula	12	14	92.3 %	92.3 %
Rectovaginal fistula	16	14	93 %	93 %
Sphincter defect	2	2	100 %	100 %

## Discussion

The main causes of RVF include obstetric or surgical trauma, inflammatory injuries (e.g., due to inflammatory bowel disease) and non-inflammatory injuries (congenital RVF). Clinically, RVF is classified into congenital and acquired types according to etiology, and injury RVF and non-injury RVF according to whether there were any injuries. In addition, the fistulas are divided into three types according to the location of the fistula opening in the vagina. High-position fistulas have a fistula opening above the rectovaginal septum and are covered by peritoneum; median-position fistulas are those with a fistula tract involving the rectovaginal septum and are located in the middle-lower section of the vagina; and low-position fistulas are those located around the dentate line. Despite the many classifications, none of these are suitable for classification in imaging.

Acquired RVF is clinically relatively rare, and the etiology is typically complex, including infections, surgery and traumas. After infection and abscess formation around the anorectal area, the abscess may penetrate the rectovaginal septum and result in RVF if not treated in a timely and proper manner. The relatively high incidence of perianal infection accounts for a slightly higher proportion of acquired RVF than other causes. In this study, 10 patients had a medical history of perianal ulceration self-rupture or incised drainage, seven patients had a history of anal cryptitis with secondary abscess formation, and five patients had a history of Bartholin’s gland abscess formation. Patients with infections accounted for 78 % of all patients. Currently, reports on the primary cause of secondary RVF are inconsistent. In Western countries, RVFs caused by birth trauma account for 88 % of all RVF cases [13] and occur in 0.1 % of all vaginal deliveries [14]. In the present study, infections were the leading cause of RVF because the anorectal disease department of Longhua Hospital specializes in treating hemorrhoids.

The clinical diagnosis of simple RVF (only one relatively short tube) based on medical history and anovaginal digital or probe examination is generally not difficult. However, for complex RVF—i.e., RVF with branches and concomitant rectovaginal septum abscesses or perianal or perineal abscesses—the diagnosis must rely on imaging examinations.

Currently, there are many imaging methods for RVF diagnosis, mainly including fistula X-rays with contrast, CT (computed tomography) imaging, ultrasonography, and MRI. MRI is the best imaging modality in many parts of the world for the diagnosis of rectovaginal fistula [15]. With MRI, the soft-tissue contrast resolution is relatively high. The anatomical structures of the internal and external anal sphincters, levator ani muscle, and puborectal muscle, as well as the relationship between the fistula and muscles surrounding the anus, can be accurately revealed. Thus, MRI has a high sensitivity for the detection of soft tissue abnormalities. MRI with phased array coils can identify most of the relatively large fistulas [16]. Endoluminal MRI further increases the signal-to-noise ratio with a relatively small visual field [17] and can provide clear images of the rectum, anal canal, vagina, and rectovaginal septum, exhibiting a higher spatial resolution than body coil imaging [18, 19]. However, due to its high costs and requirement for expensive equipment, endoluminal MRI cannot be carried out routinely at primary hospitals in China. In addition, because of the long examination time, and that metal transplantation and pacemakers are contraindications, the use of endoluminal MRI in clinical practice is limited. The diagnostic accuracies of vaginal X-ray radiography and rectum defecography are relatively low at 79 and 35 %, respectively [20, 21]. The diagnostic accuracy using the earlier generations of CT scanners was reported to be approximately 60 %, and there has been no report on the RVF diagnostic accuracy using modern multislice helical scanners [22].

**Fig. 1:**
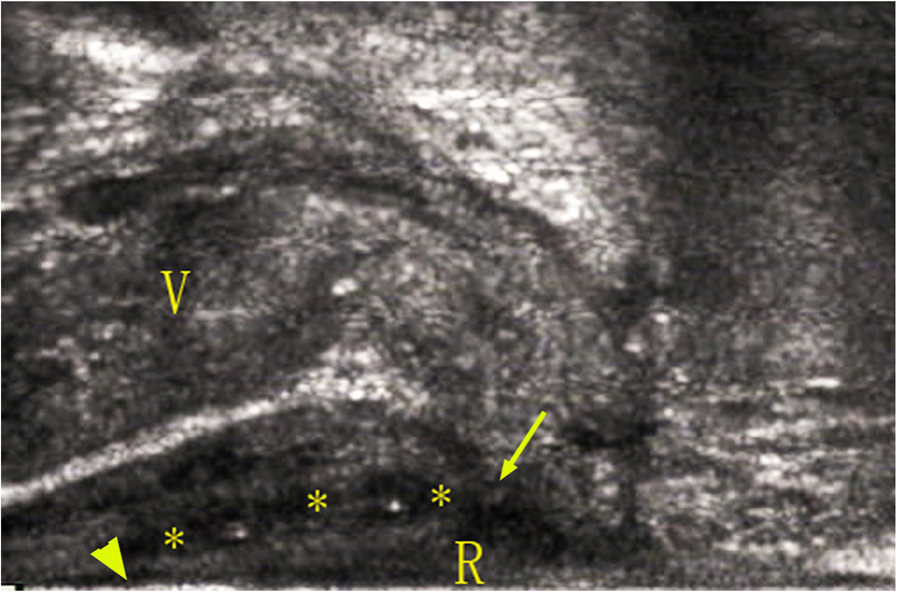
RVF morphology observed using a linear array probe on transrectal sagittal section. The ultrasonic image clearly demonstrates the fistula opening at the vagina and fistula opening in the rectal wall using the linear array probe, which is more advantageous than the convex array probe when observing fistula morphology and the surrounding anatomical structures. Abbreviations: V, vagina; R, rectum. The small arrow indicates the fistula opening at the vagina. The large arrow indicates the fistula opening in the rectal wall. The asterisks denote the fistulas, in which punctate strong echoes formed by gas can be seen

Endoluminal ultrasonography involves real-time imaging that is not disturbed by visceral movement, respiration or other factors. It can relatively clearly reveal the internal and external sphincters, making it easy for morphological classification. In addition, endosonography can clearly show the location and direction of the fistula, distribution of the branches, and location of the internal openings. Before the examination, no special patient preparation is needed. Endoluminal ultrasonography has a rather flexible application for RVF examination by going through either the rectum or vagina. Regarding the observation of the internal openings, our experience is that the image of the opening into the rectum is clearer if the probe goes through the rectum, and the image of the opening into the vagina is clearer if the probe goes through the vagina. The reason is that when the probe is closer to the vagina or rectum, it is easier to identify defects caused by the internal openings of the mucosal layer. We believe that the use of a convex array probe (i.e., axial scanning) for observation of the internal openings and sphincter damage is more advantageous than the use of a linear array probe because the surface curvature of the convex array probe is closer to the canal and rectum, and the anal canal and rectum can be fully displayed. The linear array probe is more advantageous than the convex array probe when observing fistula morphology and the surrounding anatomical structures (Fig. 1). In the present study, the positive predictive value for internal openings to the anorectum was as high as 100 %. The radius of the vaginal curvature was approximately 1.5–2 times that of the probe. The vagina could not be effectively expanded; hence, the effect of displaying the vaginal mucosal defects was poorer than that of displaying anorectal mucosal defects. There were two false-positive and two false-negative cases in detecting openings to the vagina. Re-examination of the images showed that the missing two fistulas were secondary branches with a small diameter. In the other two cases, fibrous scars were misdiagnosed as the internal opening of a branch. Thus, for internal openings located in the vagina, the positive predictive value of preoperative endosonography was 93 %, lower than that for internal openings located at the anorectum. Regarding evaluation of the direction and morphology of the tubes, the advantage of endoluminal ultrasonography is that the probe can be moved continuously for dynamic observations. Combined with axial, sagittal, and radial section scanning, all simple fistulas can be accurately displayed. For complex fistulas, misdiagnosis (false-positive or false-negative) may easily occur, especially when the complex fistulas include thin secondary branches, a lack of notable inflammation, occluded tubes, a lack of gas within the tube and thin tube walls, which may lead to a lack of obvious differences in the acoustic impedance between the fistula and surrounding soft tissues. Strong echoes generated by microbubbles are an important clue to the existence of a fistula [23]. Hence, for patients without a clear diagnosis, a contrast agent may be injected to improve the sensitivity [24] and help evaluate the scar tissue and fistula. Miguelanez et al [25] reported that, using a contrast agent, the diagnostic accuracy rate of endoluminal ultrasonography regarding the main tube was 100 % and that regarding the internal openings was 95 %.

**Fig. 2:**
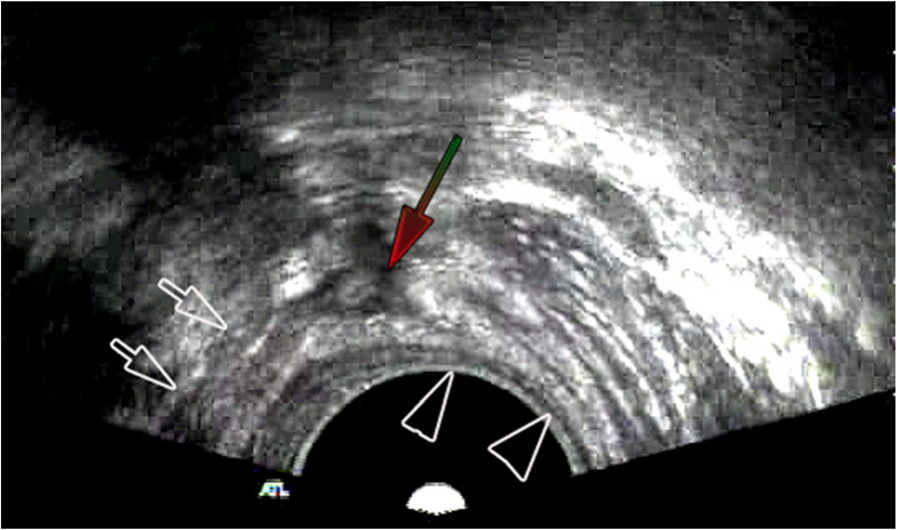
Partial continuity interruption of the external sphincter in the upper outer quadrant observed transrectally using a convex array probe. External sphincter injury with partial disarticulation of the external sphincter in the front upper outer quadrant being observed using a convex array probe, producing a clearer image than that using the linear array probe. The long arrow denotes the partially disarticulated external sphincter. The small arrow denotes the external sphincter. The large arrow denotes the internal sphincter, which is notably thinner than normal

The endosonographic manifestation of sphincter injury is as follows. Those caused by birth trauma or trauma typically show interrupted or missing muscle echoes in the external sphincter or external and internal sphincters. In severe cases, some of the normal anatomical structures, including the perineal body and rectovaginal septum, may disappear in the sonogram [26]. In patients with fecal incontinence after anal sphincter angioplasty [27], the sonogram also shows an interrupted, thin or completely internal anal sphincter because angioplasty is limited to the internal sphincter. For patients with fecal incontinence caused by primary internal sphincter degeneration [28], the sonogram only shows a substantially thinner internal sphincter. In the present study, in both cases of external sphincter injury, partial disarticulation of the external sphincter in the front upper outer quadrant was observed (Fig. 2).

Our results suggest that endoluminal ultrasonography exhibits relatively high sensitivity and positive predictive value in the diagnosis of RVF internal openings and fistula morphology. This may be related to the included cases being mostly median- or low-position fistulas caused by trauma or infection. Due to the lack of a sufficient number of cases, for secondary high-position RVF caused by tumors, radiation or inflammatory bowel disease, the conclusion would inevitably be biased. In addition, there was no true negative case in this study, presenting another drawback.

## Conclusion

In summary, diagnosing RVF using transrectal endoluminal biplane ultrasonography can not only accurately determine the internal openings in the rectum or vagina but also relatively accurately identify concomitant branches and abscesses located in the rectovaginal septum. In addition to clearly revealing the morphology of the fistula [29], endoluminal ultrasonography can also satisfactorily reveal internal and external anal sphincter injuries. To classify RVF according to the location of the fistula opening at the anal canal or rectum and fistula morphology is easy and accurate. Endoluminal ultrasonography provides useful information for both preoperative preparation and postoperative evaluation; thus, it is of relatively high clinical value.

## Availability of data and materials

Due to patient privacy protection, the data and corresponding materials of the study are only available upon individual request directed to the corresponding author.

## Abbreviations

CT: Computed tomography
MRI: Magnetic resonance imaging
RVF: Rectovaginal fistula
